# Magnesium Fertilization Improves Crop Yield in Most Production Systems: A Meta-Analysis

**DOI:** 10.3389/fpls.2019.01727

**Published:** 2020-01-24

**Authors:** Zheng Wang, Mahmood Ul Hassan, Faisal Nadeem, Liangquan Wu, Fusuo Zhang, Xuexian Li

**Affiliations:** ^1^Department of Plant Nutrition, The Key Plant-Soil Interaction Lab, MOE, China Agricultural University, Beijing, China; ^2^National Academy of Agriculture Green Development, China Agricultural University, Beijing, China; ^3^International Magnesium Institute, Fujian Agriculture and Forestry University, Fuzhou, China

**Keywords:** magnesium, crop yield, agronomic efficiency, exchangeable Mg, pH, meta-analysis

## Abstract

Magnesium deficiency is a frequently occurring limiting factor for crop production due to low levels of exchangeable Mg (ex-Mg) in acidic soil, which negatively affects sustainability of agriculture development. How Mg fertilization affects crop yield and subsequent physiological outcomes in different crop species, as well as agronomic efficiencies of Mg fertilizers, under varying soil conditions remain particular interesting questions to be addressed. A meta-analysis was performed with 570 paired observations retrieved from 99 field research articles to compare effects of Mg fertilization on crop production and corresponding agronomic efficiencies in different production systems under varying soil conditions. The mean value of yield increase and agronomic efficiency derived from Mg application was 8.5% and 34.4 kg kg^-1^ respectively, when combining all yield measurements together, regardless of the crop type, soil condition, and other factors. Under severe Mg deficiency (ex-Mg < 60 mg kg^-1^), yield increased up to 9.4%, nearly two folds of yield gain (4.9%) in the soil containing more than 120 mg kg^-1^ ex-Mg. The effects of Mg fertilization on yield was 11.3% when soil pH was lower than 6.5. The agronomic efficiency of Mg fertilizers was negatively correlated with application levels of Mg, with 38.3 kg kg^-1^ at lower MgO levels (0–50 kg ha^-1^) and 32.6 kg kg^-1^ at higher MgO levels (50–100 kg ha^-1^). Clear interactions existed between soil ex-Mg, pH, and types and amount of Mg fertilizers in terms of crop yield increase. With Mg supplementation, Mg accumulation in the leaf tissues increased by 34.3% on average; and concentrations of sugar in edible organs were 5.5% higher compared to non-Mg supplemented treatments. Our analysis corroborated that Mg fertilization enhances crop performance by improving yield or resulting in favorable physiological outcomes, providing great potentials for integrated Mg management for higher crop yield and quality.

## Introduction

Magnesium (Mg) is an essential element for crops, animals, and humans, the deficiency of which affects photosynthesis and carbohydrate partitioning in crops (Nèjia et al., 2016), reduces sustainability of agricultural production and development, and causes long-term negative impacts on human and animal health (Robert and Helen, 2004; Jeroen et al., 2015). Unfortunately, obvious symptoms of Mg deficiency frequently occur in crops, especially at their critical developmental stage with rapid carbohydrate accumulation, grown in acidic soils widely distributed across the world (Cakmak et al., 1994; Nèjia et al., 2016). Edible agricultural products are the main source of Mg nutrition for humans and animals. Therefore, maintaining Mg contents of agricultural products within relatively sufficient range is very important for animal and human health.

In an agricultural production system, the availability of Mg to crops depends on various factors such as soil texture, cation exchangeable capacity (Hariadi and Shabala, 2004), site specific climatic and anthropogenic factors, agronomic management practices, as well as crop species itself (Scheffer and Schachtschabel, 2002; Mikkelsen, 2010). Crops absorb Mg from the soil mainly through their roots. Adequate soil Mg is a key to ensure robust crop growth and production. Absolute Mg deficiency in the soil dramatically reduces Mg absorption by crop roots, which is frequently a consequence of low Mg contents in source rocks (Papenfuß and Schlichting, 1979), Mg losses by mobilization and leaching in the soil (Schachtschabel, 1954), or Mg depletion due to intensive crop production (Pol and Traore, 1993). Additionally, cationic competition, resulting from long-term imbalanced soil fertilization, causes nutrient heterogeneity in soils. A good soil Mg condition is the pre-requisite to ensure Mg uptake by crop roots and enhance Mg utilization efficiency.

Soil acidity is another important factor determining crop productivity (Mohebbi and Mahler, 1989; Aggangan et al., 1996), closely associated with deﬁciency of potassium, calcium, magnesium, phosphorus, and zinc, while toxicity of aluminum and manganese (Guo et al., 2004; Zhu et al., 2004; Binh et al., 2018) antagonizes the availability of Mg (Wang et al., 2014). In addition, the highly mobile nature of Mg^2+^ ion makes it susceptible to leaching from the root zone by heavy rainfall (Schachtschabel, 1954; Grzebise, 2011; Gransee and Führs, 2013) especially in acidic soils, reducing nutrient utilization efficiencies and crop yield.

In recent decades, more emphasis has been given to nitrogen, phosphorus, and potassium fertilizers than Mg to obtain higher crop yield (Cakmak and Yazici, 2010). Soils undergoing intensive crop forage and harvest are not being replenished with Mg fertilizers, resulting in depletion of indigenous Mg from the soil and large-scale Mg deficiency. Nowadays, Mg deficiency has become a widespread problem severely reducing photosynthetic rates of crops especially grown in acidic soils (Fischer, 1997; Sun and Payn, 1999; Ridolfi and Garrec, 2000; Graeff et al., 2001; Hermans et al., 2004). Mg deficiency symptoms typically appear on older leaves (Bergmann, 1992). Chlorosis is a most obvious response of crops to Mg deficiency that foretells considerable yield reduction as a result of decreases in sugar transport from the source to sink organs and biomass accumulation in the root and reproductive tissues (Hermans et al., 2004; Cakmak and Yazici, 2010; Gransee and Führs, 2013). From a broader point of view, Mg fertilization improves tomato yield (7.7–17.9 t ha^-1^) in South India (Kashinath et al., 2013), grain yield in barley (by 8.6%) in Iran (Mahdi et al., 2012), and hazel nut highest yield increase of 51% and total oil content increase of 4.8% in Turkey (Nedim and Daml, 2015), suggesting that Mg fertilization is an important measure to boost crop production. There is also substantial literature available on the importance of Mg for agricultural productivity, Mg deficiency in soils and crops, and Mg involvement in plant structure and physiological functions (Cakmak et al., 1994; Cakmak and Kirkby, 2008; Cakmak, 2013; Ceylan et al., 2016). However, it is imperative to better understand responses of crop yield to Mg-fertilization under different soil, cropping, and fertilization conditions in large-scale field experiments.

Until now, there has been no attempt made to systematically re-analyze effects of Mg fertilization on crop yield and agronomic efficiencies by summarizing the past experiments worldwide. Factors such as soil available Mg, soil pH, and rates and types of Mg fertilizers precondition yield responses to Mg application. In this study, a meta-analysis was conducted to (1) evaluate overall effects of Mg fertilizers on crop yield and corresponding agronomic efficiencies; (2) understand yield effects of Mg fertilization under different cropping and fertilization conditions; and (3) to estimate how exchangeable Mg and pH levels in the soil affects outcomes of Mg fertilization.

## Materials and Methods

### Search Strategy and Data Extraction

**Figure 1:**
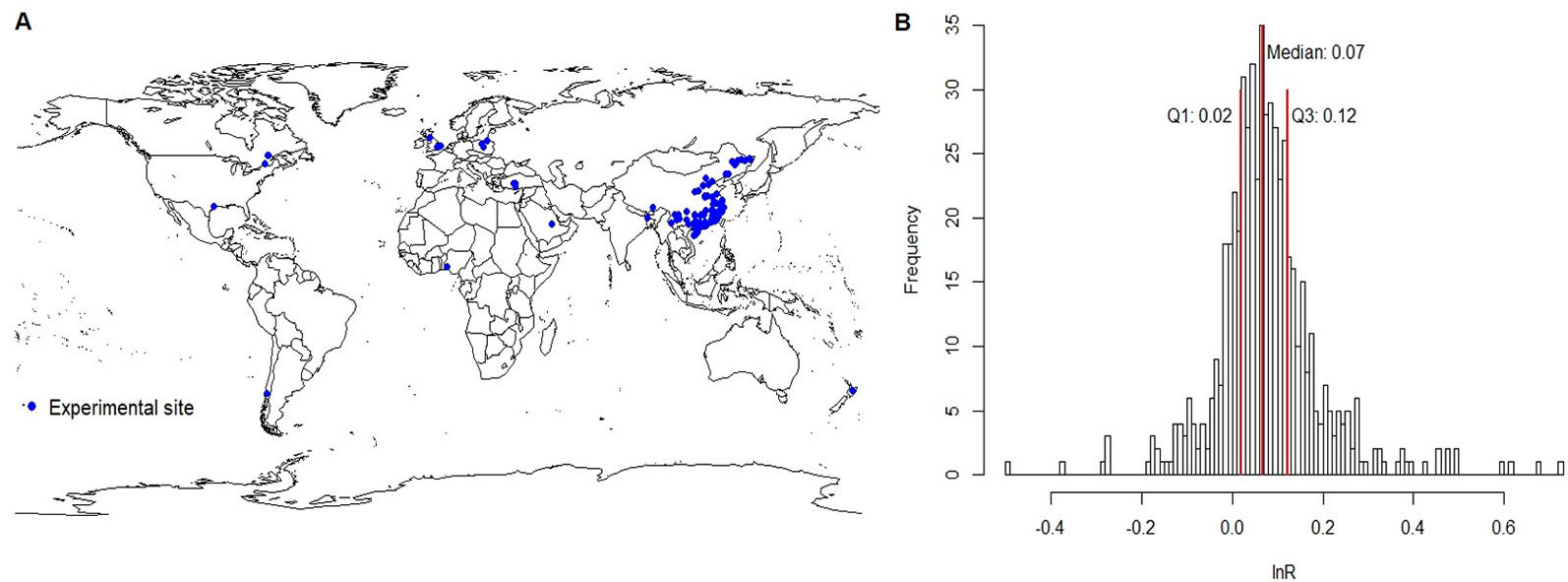
The Map distribution of experimental sites **(A)** and frequency distribution of data indicating effects of Mg fertilization on crop yield **(B)** for our meta-analysis. The blue spots indicated local experimental sites of Mg fertilizers in the field **(A)**. The three red lines of Q1 (left), Median (middle), and Q3 (right) corresponded to data frequency 25%, 50%, and 75% **(B)**.

To analyze the effect of Mg fertilizers on crop production in the field, a comprehensive literature search was performed using “Magnesium (Mg) fertiliz*,” “Magnesium (Mg) fertilis*” in the article title and “crop yield*” as key terms on Web of Science (http://apps.webofknowledge.com/) and China National Knowledge Infrastructure (http://www.cnki.net/) electronic databases before November 2019. Data were extracted either directly from tables or indirectly from conversion of original figures in reported studies including crop yield, Mg and sugar concentrations responsive to Mg fertilization around the world (**Figure 1A**; most studies from China, much less from the other countries, and no reports found from Brazil). There were very few physiological and quality data available; hence, corresponding evaluation was not included in this study. Effects of Mg fertilization on yield followed the standard normal distribution (**Figure 1B**). The studies were selected according to the following four criteria: (1) studies containing comparisons of magnesium fertilization and without magnesium fertilization (control), (2) representing field experiments, excluding pot experiment in the greenhouse, (3) with Mg fertilization in the soil, excluding foliar Mg application, (4) the study reporting types of crops, yield, the mean, and the number of paired observations (**Supplementary Figure S1**).

### Data Sources

A total of 99 papers (see study list in **Supplementary Data Sheet S1**) with 570 pairwise comparisons qualified for our meta-analysis (396 from China and 174 from other countries). The field trials were reported in ten countries (Bangladesh, Canada, China, Chile, Iran, New Zealand, Nigeria, Poland, Turkey, and United Kingdom) (**Figure 1A**).

### Effect Sizes and Their Modeling

Effects of Mg fertilization on crop yield were evaluated against corresponding control without Mg fertilization by the following equation:

$$lnR=ln(\frac{Xt}{Xc})$$

where *lnR* represented the natural log of the response ratio (the effect size), *Xt* represented the crop yield under Mg fertilization, and *Xc* represented the crop yield without Mg fertilization (Hedges et al., 1999; Verena et al., 2012). Given that more than 50% of case studies did not provide a measure of variance, case studies were weighted using numbers of study and experiment by mixed effects models in R. To interpret clearly, the effect on yield was expressed as the percentage change, which was calculated by (R−1) × 100%. A positive percentage change indicated an increase, whereas negative values indicated a decrease due to Mg fertilization. Mean percentage change was considered to be significantly different from zero if the 95% CI did not overlap with zero (Hedges et al., 1999).

Agronomic Efficiency of Mg fertilizers (AE-Mg) was calculated by the following equation:

$$AE-Mg=(Xt-Xc)/F_{Mg0}$$

where *F_MgO_* represented amount (kg MgO ha^-1^) of Mg fertilizers applied.

Statistical analysis was performed using mixed effects models in R (version 3.5.1) as follows: (1) the fixed effect, (2) the fixed effect and a random study effect, (3) the fixed effect and random effects of study and experiment nested in the study, and (4) the fixed effect and a unique experiment random effect. Appropriate random effects were identified by AIC (Akaike Information Criterion) and ANOVA analyses (R Stats Packages), with significant difference at *P* < 0.05 and *P <* 0.01 (SPSS 20.0).

### Dataset Overview

The resulting dataset contained 570 case studies, covering more than 30 crops across ten countries (**Supplementary Data Sheet S1**). According to crop characteristics and their responses to Mg fertilization, related crops were analyzed in nine groups: cereals (rice, maize, wheat, barley), fruits (apple, banana, pineapple, orange, pomelo, litchi, watermelon, sugar cane), vegetables (cabbage, lettuce, pepper, tomato, cucumber), tubers (potato, sweet potato, cassava, carrot), oil crops (soybean, peanut, canola, sunflower), grasses, tobacco, tea, and other crops (sugar beet, onion, milk thistle, blueberry).

To better interpret the results, soils were empirically divided into acidic (<6.5), neutral (6.5–7.5), and alkaline (>7.5) or Mg deficient (<60 mg kg^-1^), moderate (60—120 mg kg^-1^), and relatively sufficient (>120 mg kg^-1^) types, respectively, according to pH and exchangeable Mg levels in the soil.

Mg fertilizers were classified into two types: (1) slowly released (Mg-S) fertilizers including Mg oxide, Mg hydroxide, dolomite, Mg carbonate, and calcium-Mg phosphate, and (2) rapidly released (Mg-R) fertilizers including Mg sulfate, Mg chloride, and potassium Mg sulfate. Fertilization rates varied in a range of <50, 50–100, and >100 kg MgO ha^-1^.

## Results

### Magnesium (Mg) Fertilization Enhanced Yield of Most Crops

**Figure 2:**
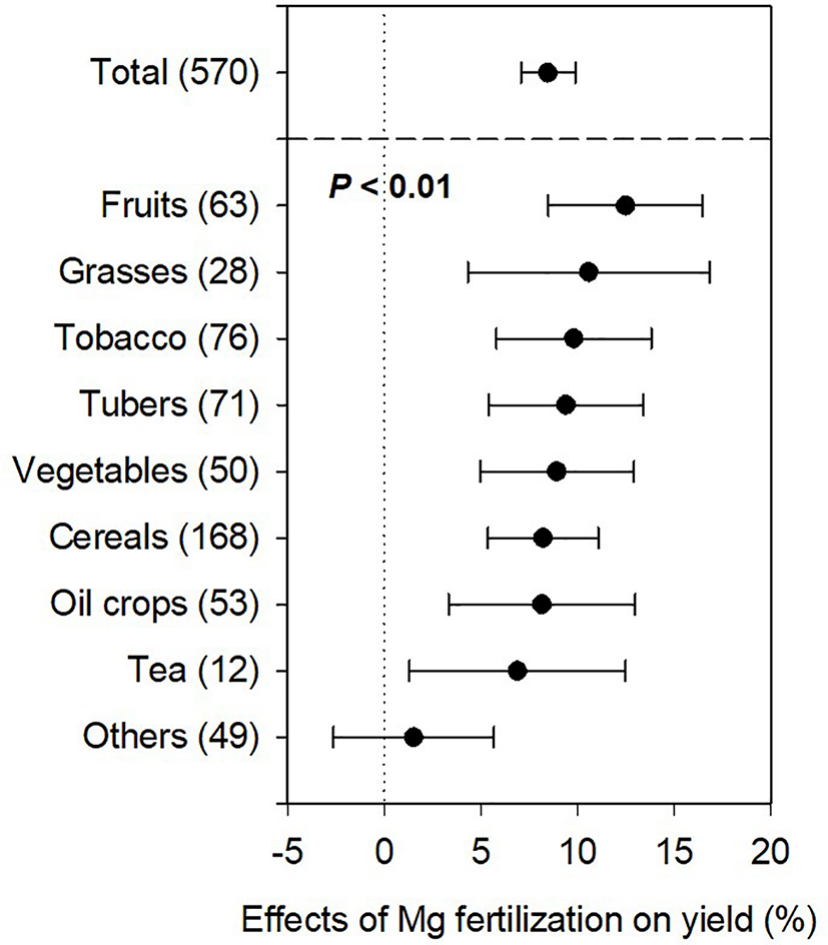
Relative effects of Mg fertilization on crop yield. The data points were means ± 95% CI (confidence interval), and the number of experimental observations were indicated in parentheses. *P*, indicated the significant differences between crops.

**Figure 3:**
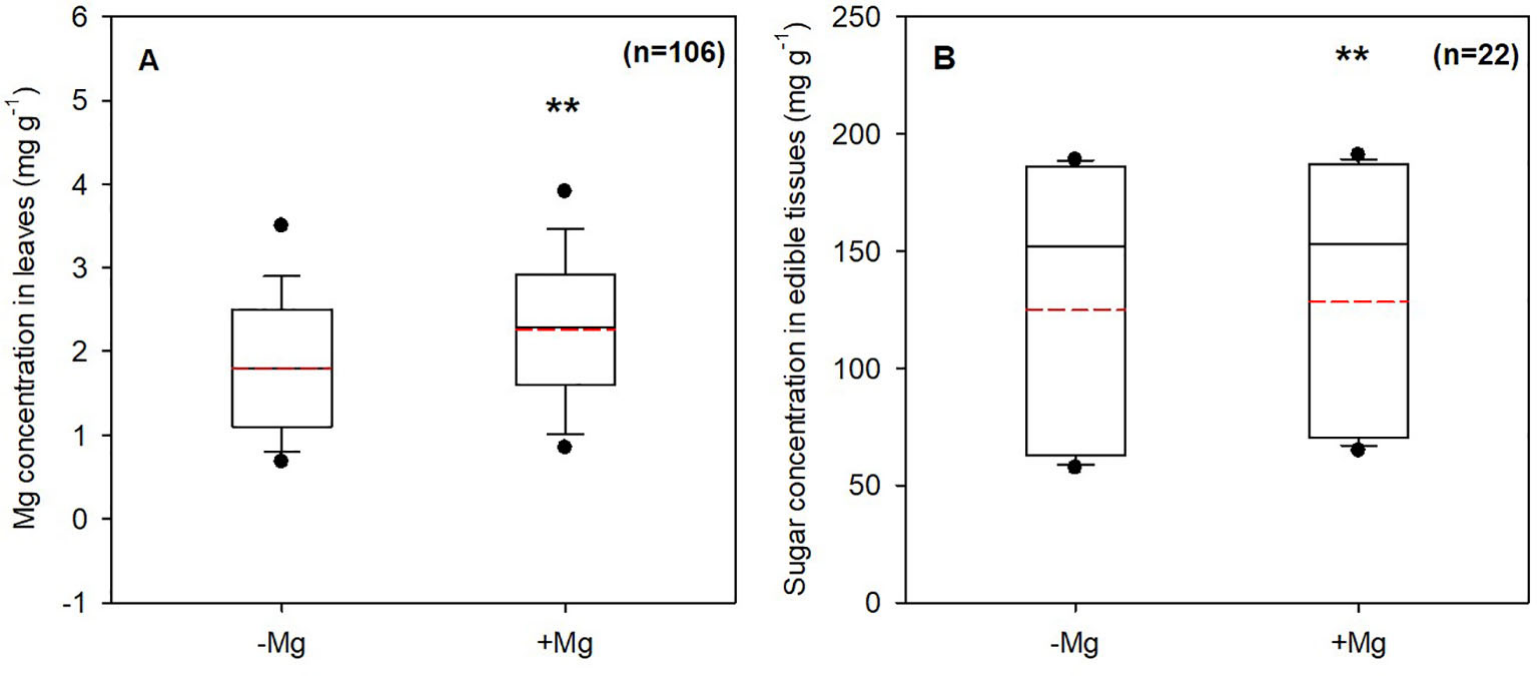
Mg in leaves **(A)** and sugar in edible tissues **(B)** concentrations with Mg (+Mg) and without Mg (-Mg) supplementation. Solid black and dashed red lines indicated the median and mean, respectively. The box boundaries indicated the 75% and 25% quartiles; error bars indicated the 90th and 10th percentiles; and the black dots indicated the 95th and 5th percentiles. **, indicated highly significant differences between treatments (*P* < 0.01).

Magnesium fertilizers generally promoted yield for most crops (**Supplementary Figure S2**) and yield increases varied depending on crop species, soil conditions, Mg fertilization rates, and other factors. The average yield increase in crop production was 8.5% according to our meta-analysis (**Figure 2**). Magnesium fertilization significantly enhanced production of fruits (12.5%), grasses (10.6%), tobacco (9.8%), tubers (9.4%), vegetables (8.9%), cereals (8.2%), oil crops (8.2%), and tea (6.9%), although non-significantly for the other crops (1.5%), compared to the non-Mg supplemented treatment at *P* < 0.05 (**Figure 2**). Moreover, average yield increases of fruit, grass, tobacco, tuber, and vegetable crops were higher than the overall average, while those of cereal, oil, tea, and other crops were lower (**Figure 2**). Crop responses to Mg differed due to soil and other related conditions. Meta-analysis revealed that Mg concentrations in leaves and sugar concentrations in crops tissues (tubers and beans) increased by 34.3% (**Figure 3A**) and 5.5% (**Figure 3B**) at *P* < 0.01, respectively, upon Mg fertilization.

### Agronomic Efficiencies of Mg Fertilizers Were Positively Correlated to Yield Increases of Most Crops

**Figure 4:**
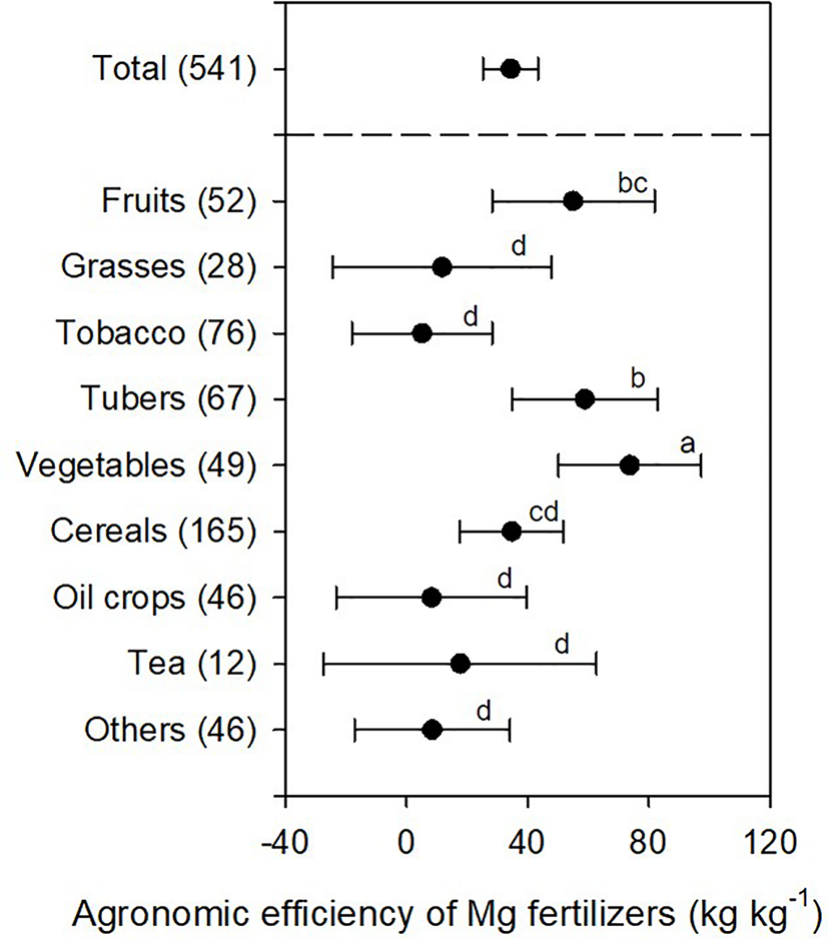
The agronomic efficiency of Mg fertilizers (AE-Mg) in different crops. The data points were means ± 95% CI (confidence interval), and the number of experimental observations were indicated in parentheses. Small letters indicated the significant differences between different crops (*P* < 0.05).

The agronomic efficiency (AE) is an important parameter indicating relative fertilization efficiency in agricultural production. AE of Mg fertilizers was defined as the yield increase per unit of Mg fertilizers applied. On average, AE-Mg was 34.4 kg kg^-1^ when 541 cases (amount of Mg fertilization was not reported in 29 cases) were combined in this study (**Figure 4**). Similar to the effect of crop species on yield increases, the agronomic efficiencies of Mg fertilizers (AE-Mg) was also affected by crop species, though in a manner inconsistent with the former effect. The AE-Mg of vegetable (73.7 kg kg^-1^) was significantly higher than tuber (58.8 kg kg^-1^), fruit (55.0 kg kg^-1^), and cereal (34.7 kg kg^-1^) crops at *P* < 0.05 (**Figure 4**). However, there was no significant difference in the AE-Mg between tea, grasses, oil, tobacco, and other crop experiments due to large variations (**Figure 4**).

**Figure 5:**
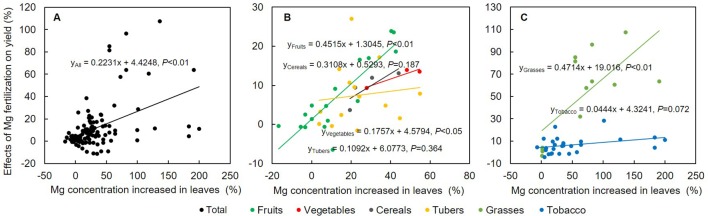
The relationship between effects of Mg fertilization on yield and variations in Mg concentrations in all crops **(A)**, vegetables, tubers, fruits, cereals **(B)**, grasses and tobacco **(C)**. Individual crop was represented by colored circle, and the response relation is fitted by a straight line of the same color line. *P*-value, indicated the significance of the results.

**Figure 6:**
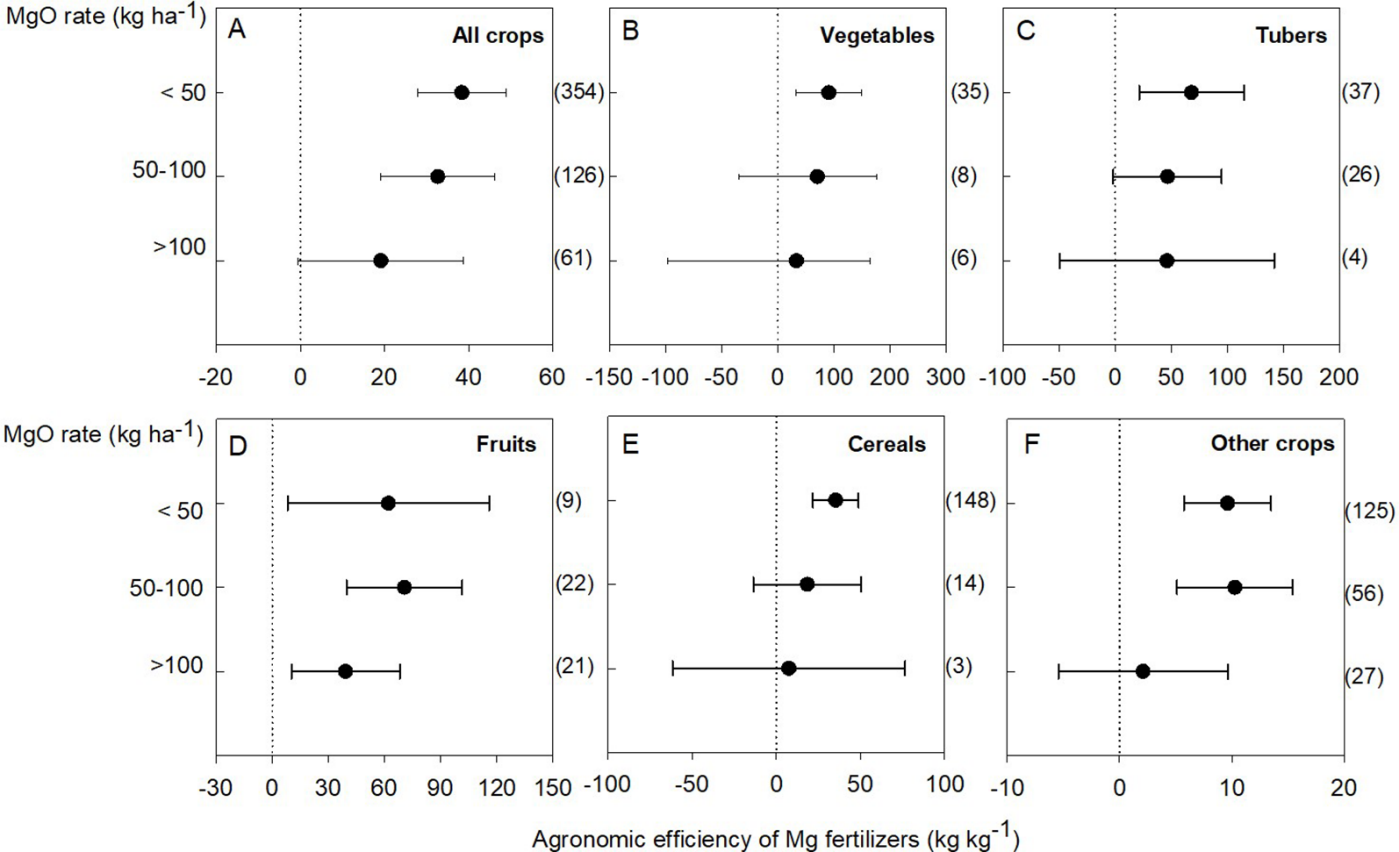
Agronomic efficiency of Mg fertilizers (AE-Mg) in all crops **(A)**, vegetables **(B)**, tubers **(C)**, fruits **(D)**, cereals **(E)**, and other crops (tobacco, tea, grasses, oil, and other crops) **(F)**. The data points were means ± 95% CI (confidence interval), and the number of experimental observations were indicated in parentheses. MgO, magnesium oxide.

AE-Mg calculation was based on fresh weights of harvested parts of different crops (except dry matter yield for grasses). Higher water content in the harvested organ tended to increase AE-Mg. Responses of crops to Mg (**Figure 5**) and the amount of Mg fertilizers applied (**Figure 6**) also affected the AE-Mg. Among four types of crops (vegetables, tubers, fruits, and cereals) responsive to Mg fertilization (**Figure 4**), yield increases in vegetables (*P* < 0.05) and fruits (*P* < 0.01) had significant correlation with Mg concentrations (**Figure 5B**).

Generally, the AE-Mg responded to Mg application when lower than 100 kg MgO ha^-1^ was applied (**Figure 6A**). Although there was no data for sugarcane (in the fruits group) and sugar beet (in the other crops group) under Mg fertilization lower than 50 kg MgO ha^-1^, the AE-Mg in vegetable (90.8 kg kg^-1^), tuber (68.0 kg kg^-1^), and cereal (35.3 kg kg^-1^) crops was responsive to Mg fertilization lower than 50 kg MgO ha^-1^ (**Figures 6B, C, E**); the AE-Mg in fruit (62.0 kg kg^-1^) (**Figure 6D**) and other crops (9.6 kg kg^-1^) (**Figure 6F**) was responsive even in the range of 50–100 kg MgO ha^-1^. Notably, fruit crops responded to Mg application higher than 100 kg MgO ha^-1^ (**Figure 6E**). The difference was probably due to differential responses of crops to Mg, which conferred yield variations in relation to concentration changes of Mg in leaves (**Figure 5**). Importantly, there was a significant positive liner correlation between the crop yield and Mg concentration in leaves (*P* < 0.01, **Figure 5A**). With regard to different crop categories, the linear correlation was significant for vegetables (*P* < 0.05), fruits, and grasses (*P* < 0.01) (**Figures 5B, C**).

### Soil Conditions and Fertilizer Types Affected Fertilization Effects

**Figure 7:**
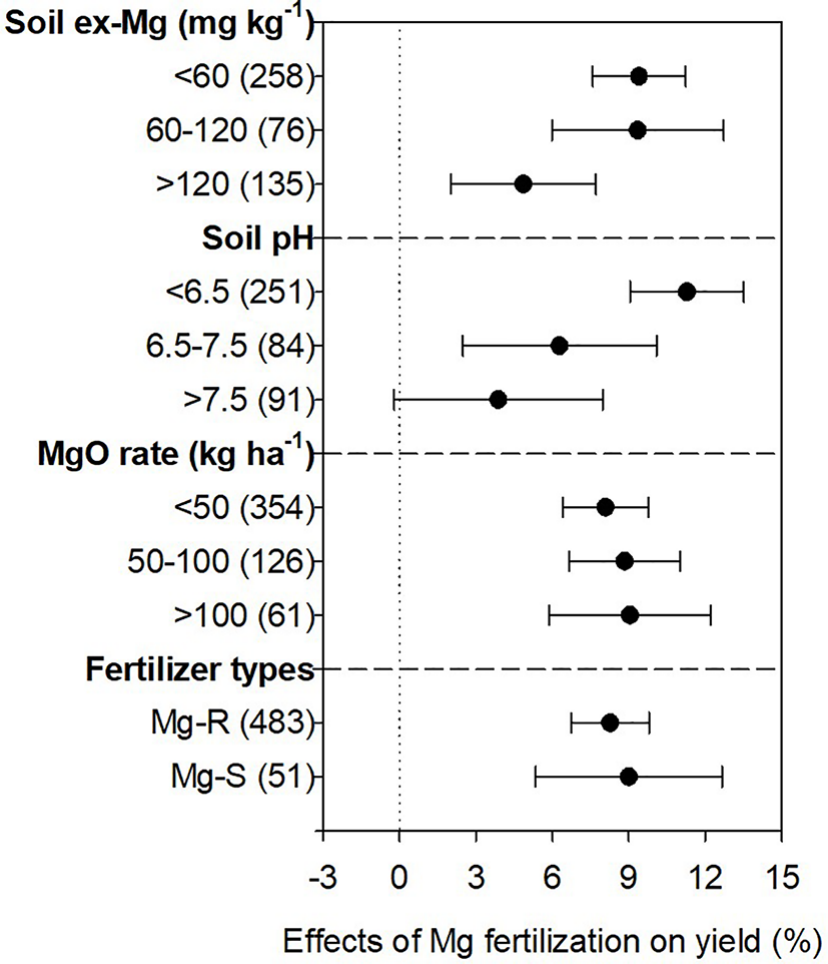
Effects of Mg fertilizer on crop yield under different soil conditions (exchangeable-Mg concentrations, soil pH, rates of MgO application, and types of Mg fertilizers). The data points were means ± 95% CI (confidence interval), and the number of experimental observations were indicated in parentheses. Soil ex-Mg, soil exchangeable magnesium; MgO, magnesium oxide; Mg-R, rapidly released Mg fertilizers; Mg-S, slowly released Mg fertilizers.

Crop roots explore heterogeneously available mineral nutrients in the soil for absorption to sustain plant growth and development (Hodge, 2004; Nibau et al., 2008). Soil conditions, e.g. concentrations of exchangeable Mg and soil pH levels, have a direct effect on Mg availability to crops thereby affecting crop yield in the long run (Foy and Barber, 1958; Fox and Piekielek, 1984; Clarka et al., 1997). Our meta-analysis suggested obvious stimulatory effects of Mg fertilization on crop yield in Mg-deficient acidic soils (**Figure 7**). Crop yield increased by 9.4%, 9.4%, and 4.9% due to Mg fertilization respectively under Mg deficient (exchangeable Mg <60 mg kg^-1^), moderate (60–120 mg kg^-1^), and relatively sufficient (> 120 mg kg^-1^) conditions. Similarly, Mg improved crop production by 11.3%, 6.3%, and 3.9% respectively under acid (pH <6.5), neutral (pH 6.5–7.5), and alkaline (pH >7.5) soil conditions (**Figure 7**). Yield increases were positively correlated with the amount of Mg fertilizers especially at application levels higher than 100 kg MgO ha^-1^ (9.0% yield-increment, **Figure 7**). Nevertheless, two different types of Mg fertilizers Mg-R (8.3%) and Mg-S (9.0%) showed no significant difference in yield improvement (**Figure 7**).

### Interaction Effects of Ex-Mg and Fertilization Rates, Ex-Mg and pH, and pH and Fertilizer Types

**Figure 8:**
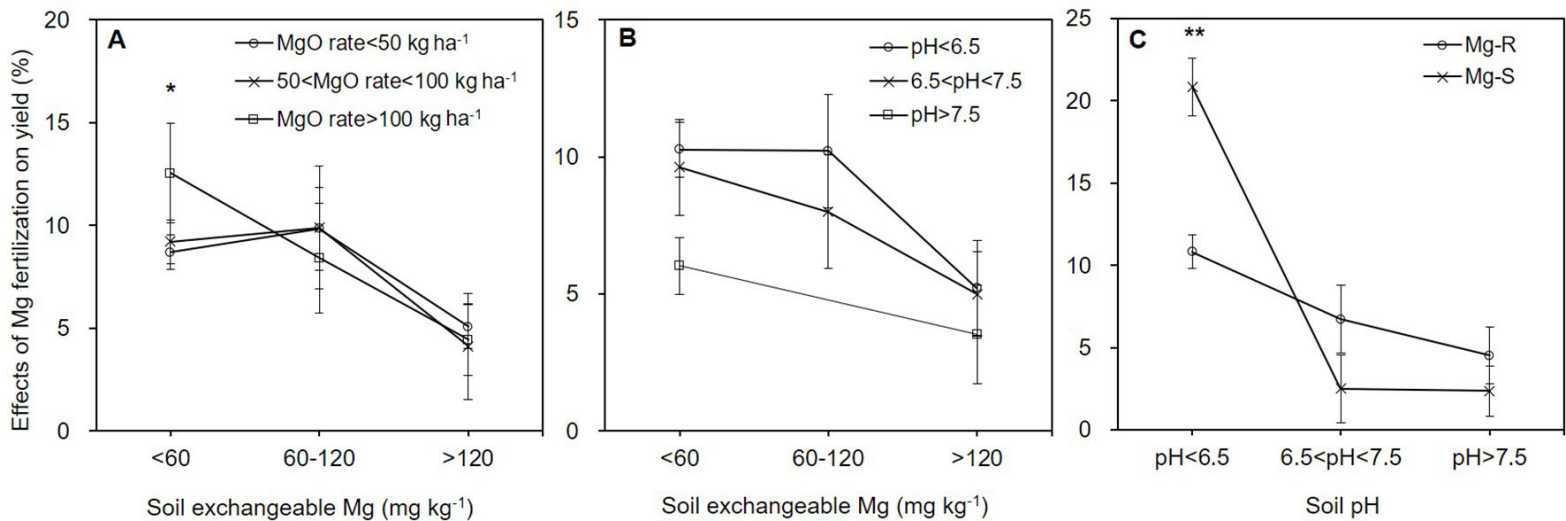
Interaction effects of two factors on yield increases: soil exchangeable Mg and rates of Mg fertilizers **(A)**, soil exchangeable-Mg and pH **(B)**, soil pH and Mg fertilizer types **(C)**. * and **, indicated significant differences at *P* < 0.05 and *P* < 0.01, respectively. MgO, magnesium oxide; Mg-R, rapidly released Mg fertilizers; Mg-S, slowly released Mg fertilizers.

Given large variations in fertilization regimes and soil conditions in field experiments, it's necessary to evaluate interaction effects of different influential factors on stimulatory effects of Mg fertilization on yield. The ex-Mg level was the significant factor compared with application rates of Mg fertilizers (*P* < 0.05, **Table S1**). With exchangeable-Mg concentrations in the soil increasing, crop yield responded moderately or slightly to Mg fertilization. Notably, Mg application higher than 100 kg MgO ha^-1^ in Mg deficient soils gave rise to the largest yield gain (12.5%) (**Figure 8A**). Adjustment of MgO rates caused no significant difference in soils with moderate or relatively sufficient ex-Mg (**Figure 8A**).

Indeed, the effect of Mg-fertilizers on crop production was combinatorically determined by pH levels and ex-Mg status of soils (*P* = 0.803, **Supplementary Table S2**), with the ex-Mg concentration as a main influential factor (*P* = 0.05, **Supplementary Table S2**). Average yield increases derived from Mg-fertilization under Mg deficiency were greater than those under moderate or relatively sufficient Mg conditions regardless of variations in soil pH (**Figure 8**). However, the interaction effect of soil pH and Mg-fertilizer types was significant (*P* < 0.05, **Supplementary Table S3**). The Mg-S type significantly improved crop yield (20.9%) compared to the Mg-R type (10.8%) in acidic soils (*P* < 0.01, **Figure 8C**). Mg-S type also has a certain effect on improving soil acidity, which indirectly improves the utilization efficiency; and Mg-R performed better than Mg-S in neutral and alkaline soils (**Figure 8C**).

## Discussion

### Magnesium Application Increases Crop Yield

Magnesium plays essential roles in ensuring crop productivity (Senbayram et al., 2015); unfortunately, Mg concentration in wheat, fruits, and vegetables has declined over the past 50 years (Andrea, 2013). Latent and acute Mg deficiencies are common phenomena in crop production (Römheld and Kirkby, 2007). Magnesium fertilization improves crop yield in the field (Mahdi et al., 2012; Kashinath et al., 2013; Nedim and Daml, 2015). Given large variations in crop species, fertilization regimes, and soil and climatic conditions in field experiments, it's necessary to systemically evaluate or quantify the overall effects of Mg fertilization on crop yield, corresponding agronomic efficiencies, and how pH and exchangeable Mg levels influence effects of Mg fertilization. Here, we selected 396 sets of observations from China and 174 outside of China to analyze how soil application of Mg fertilizers affect crop production in the field.

Our meta-analysis showed higher yield in fruit, grass, tobacco, tuber, vegetable, cereal, oil crop, tea, and other crops production with an overall 8.5% increase (**Figure 2**) when reasonable amount of Mg (i.e., 94.1, 46.9, 54.1, 58.3, 43.5, 27.8, 47.2, 34.1, and 76.8 kg MgO ha^-1^, respectively) was applied. Under Mg deficiency, Mg fertilization leads to large yield increases; when not deficient, applied Mg meets high demand of crops during their rapid growth period. Alternatively, high concentrations of ions such as K^+^, Ca^2+^, and NH_4_^+^ likely antagonize Mg^2+^ uptake (Mulder, 1956; Seggewiss and Jungk, 1988; Wilkinson et al., 1990; Marschner, 2012); therefore, Mg fertilization upscales the Mg^2+^ proportion and weakens other cationic antagonism in the soil solution. Magnesium deficiency hampers nutrient uptake and reduces the leaf growth rate, affecting the assimilate supply to growing roots and their capacity to acquire nutrients and ultimately decreases the yield (Cakmak and Kirkby, 2008).

Magnesium is key component of several biological processes (CO_2_ fixation in photosynthesis, photophosphorylation, protein and chlorophyll synthesis, phloem loading, and translocation of assimilates) in leaves (Cakmak and Yazici, 2010). The photosynthetic assimilates from leaves are transported to the sink organs (such as roots, shoot tips, and seeds), and stored as starch or converted to hexoses (Cakmak et al., 1994; Hermans et al., 2005; Lemoine et al., 2013) to increase crop yield under sufficient Mg status (Brohi et al., 2000; Laing et al., 2000). Sucrose transport from source to sink tissues occurs through phloem by invertase and sucrose synthase enzymes (Sturm and Tang, 1999; Winter and Huber, 2000; Welham et al., 2009). Hence, appropriate Mg concentration in leaves is essential to ensure activities of enzymes involved in source-to-sink transport of Mg and sugars, which can be achieved by planting proper species as well as managing Mg fertilizer rates (White and Broadley, 2009).

Mg^2+^ and closely related sugar production in leaves are of utmost importance for biomass accumulation and grain development (Koch, 1996; Orlovius and McHoul, 2015). Mg^2+^ also promotes assimilate partitioning and translocation to source tissues (Cakmak and Kirkby, 2008; Cakmak, 2013). Mg-deficiency reduces grain weight and lowers grain quality in wheat (Ceylan et al., 2016). We found that sugar concentrations in crops increased when Mg was applied compared to those without Mg application (**Figure 3B**). Enhanced sugar accumulation due to Mg fertilization is beneficial for crop production, regardless of plant species (Strebel and Duynisveld, 1989; Marschner, 2012; Orlovius and McHoul, 2015).

### Agronomic Efficiencies of Mg Fertilizers Varies Depending on Crop Species

Mg^2+^ plays a critical role in regulating photosynthesis (Sun and Payn, 1999); Mg deficiency severely down-regulates photosynthesis rates, photo assimilates transport to sinks and crop yield (Nèjia et al., 2016). Magnesium application promoted Mg concentration in leaves (**Figure 3A**) and crop yield (**Figure 2**). The increased Mg concentration in leaves favored yield increases in all crops (**Figure 5A**) and significant responses were observed in fruits (*P* < 0.01), vegetables (*P* < 0.05) (**Figure 5B**), and grasses (*P* < 0.01, **Figure 5C**). However, the agronomic efficiencies of Mg fertilizers (AE-Mg) showed a different pattern due to variations in uptake or utilization of Mg across crop species (**Figure 4**). We analyzed 541 dataset and identified the AE-Mg as 34.4 kg kg^-1^ on average (**Figure 4**). Vegetables were always most responsive to Mg application, and cereals were least responsive (**Figure 4**). Even for cereals, the AE-Mg was 34.7 kg kg^-1^ (**Figure 4**), dramatically higher than that of nitrogen (8.0–10.4 kg kg^-1^), phosphorus (7.3–9.0 kg kg^-1^), and potassium (5.3–6.3 kg kg^-1^) (Zhang et al., 2008). Plants generally have similar concentrations of Mg and P (Marschner, 2012); However, in contrast to long-term NPK fertilization, Mg removal from the soil by crop harvest has not been supplemented and Mg is more easily leached (Schachtschabel, 1954; Grzebise, 2011; Gransee and Führs, 2013), resulting in larger yield effects and higher AE-Mg upon Mg application.

### Soil Conditions Primarily Determine Yield Effects of Mg Fertilization

Soil pH directly affects magnesium release from clay minerals and Mg uptake by plants (Schubert et al., 1990). Exchangeable Mg at pH <6.0 becomes non-exchangeable when soil pH becomes higher than 6.5 (Chan et al., 1979; Hailes et al., 1997). Mg is subjected to leaching in acidic soils, and H^+^, Al^3+^, and Mn^2+^ in rhizosphere may interfere with Mg uptake, thus hampering crop yield (Metson, 1974; Mayland and Wilkinson, 1989). Mg fertilization not only increases bioavailability of Mg^2+^, but also mitigates Al^3+^ and Mn^2+^ toxicity (Bot et al., 1990; Goss and Carvalho, 1992; Bose et al., 2011; Marschner, 2012). Therefore, dramatic yield increases were observed when exchangeable Mg was lower than 60 mg kg^-1^ or pH was below 6.5, with less extent of yield effects under other conditions (**Figure 7**). Crops cultivated on Mg deficient soils show positive responses to the applied Mg fertilizers depending on the rate and timing of application (White and Broadley, 2009; Grzebisz, 2013). Thus, the application of Mg fertilizer in the acidic and Mg deficient soil is very important for crop nutrient management.

The yield effect was the largest in the magnesium deficient soil irrespective of MgO rates (**Figure 8A**) and soil pH (**Figure 8B**). Although exchangeable-Mg levels were the primary factors determining yield increases (**Supplementary Tables S1** and **S2**), there were clear interactions between soil pH and fertilizer types (**Supplementary Table S3**). Mg fertilizers are generally classified into rapidly released (Mg-R) and slowly released (Mg-S) types with distinct particle size and water solubility (Mayland and Wilkinson, 1989; Härdter et al., 2004; Loganathan et al., 2005). Mg-S releases slowly and improved yield more efficiently as compared to Mg-R (**Figure 8C**). Mg-S is also efficiently absorbed by crops and neutralizes soil acids. Both Mg-R and Mg-S improved crop yield with no significant difference between two types of Mg fertilizers (**Figure 8**).

## Conclusions

Magnesium has similar concentrations to phosphorus in plant tissues. However, Mg is easily leached out in acidic soils and competition of excessive cations makes Mg less available to plant roots. Unfortunately, Mg deficiency is not well aware by farmers. Thus, Mg limitation is becoming an increasingly severe limitation factor in crop production. Our analysis suggested that Mg application improved crop yield by 8.5% under various field conditions across the world, along with elevation of Mg and sugar concentrations in plant tissues. The yield increase was 10.6% under severe Mg deficiency and 10.8% when soil pH was lower than 6.5.

The agronomic efficiency of magnesium fertilizers was 34.4 kg kg^-1^ and increased up to 38.3 kg kg^-1^ at lower MgO levels (0–50 kg ha^-1^), which is dramatically higher than that of nitrogen, phosphorus, and potassium. Our findings indicate that it is more efficient in terms of yield improvement by applying Mg fertilizers compared to application of other macronutrients, opening up a novel path towards high nutrient efficiency, balanced fertilization for high crop yield and quality, as well as sustainable development of agriculture.

## Author Contributions

XL and FZ designed research. ZW, MH, FN, and LW collected data. ZW and XL wrote the paper. FZ revised the manuscript. All authors approved the final manuscript.

## Funding

This work was funded by the International Magnesium Institute (IMI, Fujian Agriculture and Forestry University, China) and Chinese National Basic Research Program (2015CB150400).

## Conflict of Interest

The authors declare that the research was conducted in the absence of any commercial or financial relationships that could be construed as a potential conflict of interest.

## Abbreviations

N, nitrogen; P, phosphorus; K, potassium; Ca, calcium; Mg, magnesium; AE-Mg, agronomic efficiency of Mg fertilizers; ex-Mg, exchangeable magnesium.

## Supplementary Material

Supplementary Data Sheet S1 Study list used for meta-analysis.
