# Supplementary material for: Magnesium Fertilization Improves Crop Yield in Most Production Systems: A Meta-Analysis
Source: Front Plant Sci. 2020 Jan 24;10:1727. doi: 10.3389/fpls.2019.01727 (PMC6992656; doi:10.3389/fpls.2019.01727)
Supplement: Supplementary file 2 [file Image_1.pdf]

Supplementary Figure S1

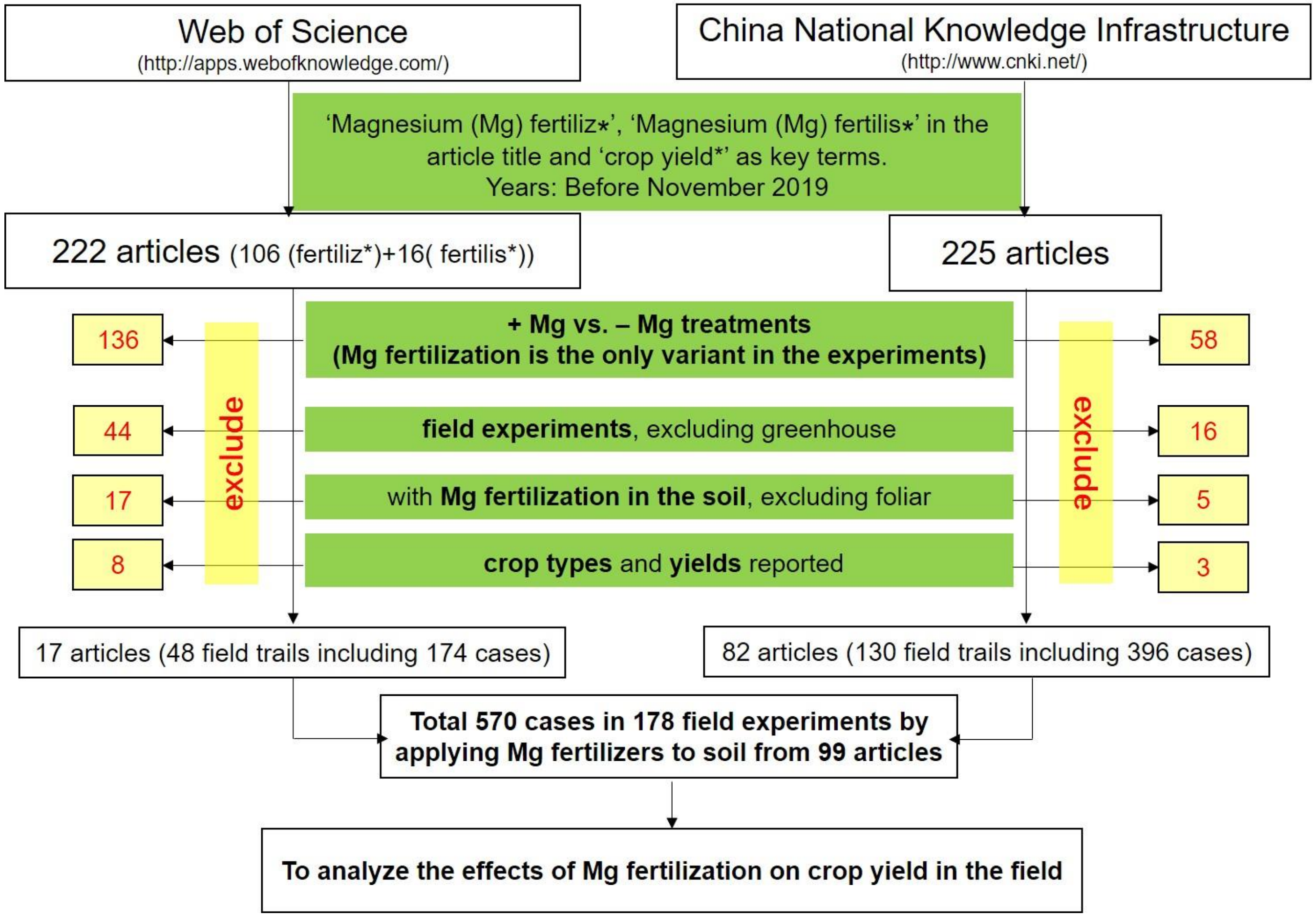

Figure S1 | Screening method of Mg dataset by preferred reporting items for systematic reviews and Meta-analysis (PRISMA).
