# Supplementary material for: Magnesium Fertilization Improves Crop Yield in Most Production Systems: A Meta-Analysis
Source: Front Plant Sci. 2020 Jan 24;10:1727. doi: 10.3389/fpls.2019.01727 (PMC6992656; doi:10.3389/fpls.2019.01727)
Supplement: Supplementary file 3 [file Image_2.pdf]

Supplementary Material

Supplementary Figure S2

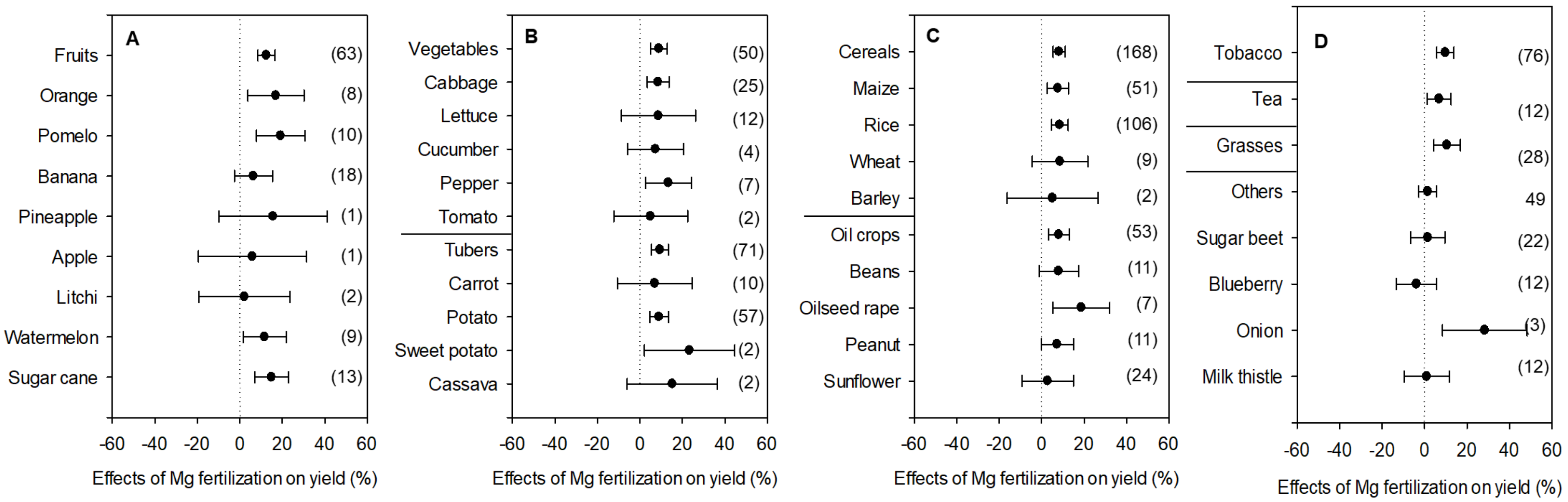

Figure S2 | Effects of Mg fertilization on yield (crop groups based on figure 2) of fruits **(A)**, vegetables and tubers **(B)**, cereals and oil crops **(C)** tobacco, tea, grasses and other crops **(D).** The data points were means ± 95% CI (confidence interval), and the number of experimental observations were indicated in parentheses.
