# Supplementary material for: Magnesium Fertilization Improves Crop Yield in Most Production Systems: A Meta-Analysis
Source: Front Plant Sci. 2020 Jan 24;10:1727. doi: 10.3389/fpls.2019.01727 (PMC6992656; doi:10.3389/fpls.2019.01727)
Supplement: Supplementary file 4 [file Table_1.pdf]

Supplementary Material

Supplementary Table S1

Table S1 Analysis of interaction effects between soil exchangeable Mg and MgO rates for yield improvement

Source of Variation	SS	df	MS	F	*P*
S_1 (Soil ex-Mg)	1650.60	2	825.30	6.246	0.002**
S_2 (MgO rates)	2.25	2	1.12	0.009	0.992
Interaction (S_1*S_2)	574.67	4	143.67	1.087	0.362
Error	58142.76	440	132.14		
Total	89390.16	449			

Soil ex-Mg, soil exchangeable magnesium concentration; MgO rate, the amount of MgO in applied magnesium fertilizer. **, significance at $P < 0.01$
