# Supplementary material for: Magnesium Fertilization Improves Crop Yield in Most Production Systems: A Meta-Analysis
Source: Front Plant Sci. 2020 Jan 24;10:1727. doi: 10.3389/fpls.2019.01727 (PMC6992656; doi:10.3389/fpls.2019.01727)
Supplement: Supplementary file 5 [file Table_2.pdf]

Supplementary Table S2

Table S2 Analysis of interaction effects between soil pH and soil ex-Mg for yield improvement

Source of Variation	SS	df	MS	F	*P*
S_2 (Soil ex-Mg)	844.89	2	422.45	2.915	0.050*
S_1 (Soil pH)	444.85	2	222.43	1.535	0.217
Interaction (S_1*S_2)	144.13	3	48.04	0.332	0.803
Error	54489.91	376	144.92		
Total	83838.56	384			

Soil ex-Mg: soil exchangeable Mg concentration. *, significance at $P < 0.05$
