# Supplementary material for: Magnesium Fertilization Improves Crop Yield in Most Production Systems: A Meta-Analysis
Source: Front Plant Sci. 2020 Jan 24;10:1727. doi: 10.3389/fpls.2019.01727 (PMC6992656; doi:10.3389/fpls.2019.01727)
Supplement: Supplementary file 6 [file Table_3.pdf]

Supplementary Table S3

Table S3 Analysis of interaction effects between soil pH and Mg fertilizer types for yield improvement

Source of Variation	SS	df	MS	F	*P*
S_1 (Soil pH)	5783.01	2	2891.51	13.163	0.000**
S_2 (Mg types)	47.07	1	47.07	0.214	0.644
Interaction (S_1*S_2)	1777.16	2	888.58	4.045	0.018*
Error	90720.07	413	219.66		
Total	134663.44	419			

* and **, significance at $P < 0.05$ and $P < 0.01$, respectively.
